# Experimental Investigations into Using Motion Capture State Feedback for Real-Time Control of a Humanoid Robot

**DOI:** 10.3390/s22249853

**Published:** 2022-12-15

**Authors:** Mihaela Popescu, Dennis Mronga, Ivan Bergonzani, Shivesh Kumar, Frank Kirchner

**Affiliations:** 1 Robotics Group, Faculty of Mathematics and Computer Science, University of Bremen, 28359 Bremen, Germany; 2 Robotics Innovation Center, German Research Center for Artificial Intelligence (DFKI GmbH), 28359 Bremen, Germany

**Keywords:** humanoid robot, state estimation, motion capture, Whole-Body Control

## Abstract

Regardless of recent advances, humanoid robots still face significant difficulties in performing locomotion tasks. Among the key challenges that must be addressed to achieve robust bipedal locomotion are dynamically consistent motion planning, feedback control, and state estimation of such complex systems. In this paper, we investigate the use of an external motion capture system to provide state feedback to an online whole-body controller. We present experimental results with the humanoid robot RH5 performing two different whole-body motions: squatting with both feet in contact with the ground and balancing on one leg. We compare the execution of these motions using state feedback from (i) an external motion tracking system and (ii) an internal state estimator based on inertial measurement unit (IMU), forward kinematics, and contact sensing. It is shown that state-of-the-art motion capture systems can be successfully used in the high-frequency feedback control loop of humanoid robots, providing an alternative in cases where state estimation is not reliable.

## 1. Introduction

Humanoid robots are complex systems, both in terms of modeling and control. Bipedal locomotion is particularly difficult due to the instability of the robot in walking phases with double or single ground contacts. Balance is highly dependent on the control approaches employed and the accuracy of the floating base state estimation. The latter is commonly achieved using onboard sensors, which are subject to drift and noise. In contrast, external tracking approaches provide a globally stable estimate of the robot’s state, independent of inertial sensor drift and kinematic modeling errors such as leg flexibility.

Marker-based motion capture systems (MoCap) have been used for various robotic applications. One widely explored topic is human motion imitation. Motion data acquisition enables humanoid robots to perform human-like movement sequences such as walking and dancing. For instance, ref. [1] proposes a trajectory generation method for humanoid robots to imitate human walking gaits captured with a marker-based motion capture system. The human movements are adapted to match the kinematic structure, degrees of freedom, and joint limits of the humanoid robot. The work in [2] presents the use of human motion data to generate natural walking and turning motions on the humanoid robot HRP-4C, while considering dynamic balance. Moreover, the work of [3] addresses the topic of human–robot interaction by generating human-like locomotion trajectories for the humanoid robot TALOS. Motion capture is used to compare the computed robot trajectories with previously recorded human walking trajectories and to evaluate which walking pattern generation model is more realistic. Dancing motion generation is a challenging task as well, since it often requires quicker motions than walking. The work in [4] proposes a control approach, which enables the HRP-2 humanoid robot to perform human-like dancing based on motion capture data, while maintaining balance and enforcing actuation limits.

Another important application of motion capture systems is state estimation of mobile robots. Very often, MoCap is used as precise ground truth to determine the position and orientation of the floating base of a humanoid robot. For example, MoCap has been used to evaluate different state estimation approaches based on proprioceptive sensors [5], LiDAR and kinematic-inertial data fusion [6], as well as LiDAR fused with visual-inertial odometry [7]. Less frequently, external motion capture systems have been used to provide state feedback to the control loops of legged robots. The LittleDog quadruped robot [8] uses a set of retroreflective markers placed both on the robot body and legs as well as on the terrain surface to allow analysis of different locomotion strategies without robot perception. The motion capture runs at a low frequency of approximately 100 Hz since the speed of state estimation is not crucial for the balance of the robot with four contact points. The hopping leg Salto-1P [9] executes precise hopping by using motion capture data to estimate the position and orientation of the robot. However, the motion capture has a low frequency of 100 Hz, which is not sufficient for a fast feedback control loop and requires an additional Kalman filter for position estimation and fusion with the gyroscope readings from the onboard IMU for attitude estimation. Moreover, marker-based motion capture has been used to track the position and orientation of the HRP-2 humanoid robot’s chest, while performing locomotion and pulling a fire hose, which acts as an external force on the robot’s wrist [10]. The tracked robot pose is used as input to the walking pattern generator to correct the orientation drift and improve the robot’s balance during locomotion. However, the success rate of the HRP-2 humanoid robot experiments is only 50%, and the motion capture system has a low acquisition frequency of 200 Hz.

In this work, we present an experimental study to demonstrate for the first time that it is possible to employ a high-frequency motion capture system for online stabilization of the humanoid robot RH5 [11]. We use motion capture as an alternative for proprioceptive state estimation. The whole-body controller in [12] is used to stabilize legged motions such as squats and balancing on one leg.

In particular, the paper presents (i) the usage of a high-frequency marker-based motion capture framework for robot floating base tracking, (ii) Whole-Body Control of the humanoid robot with motion capture position feedback, (iii) the experimental validation of the proposed approach with the humanoid robot RH5 performing squats and balancing on one leg and (iv) a comparison with a state estimation approach [13] based on internal sensor measurements, namely IMU, leg kinematics and foot contact sensors. We believe that motion capture can be a viable alternative to state estimation to address edge cases of humanoid locomotion where state estimation is not reliable. As an alternative, the two approaches could potentially be combined.

This paper is organized as follows. In Section 2, we describe the motion capture framework and the Whole-Body Control algorithm. Section 3 presents the experimental results of squatting and single leg balancing of the humanoid robot RH5. In Section 5, we draw the conclusions and propose future research directions.

## 2. Materials and Methods

**Figure 1:**
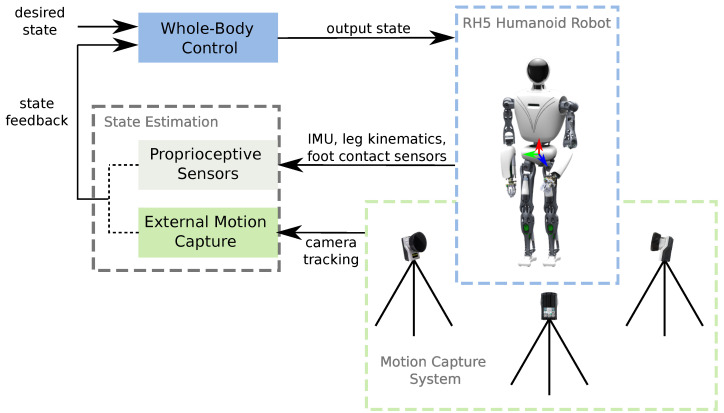
The control architecture of the humanoid robot RH5 includes a Whole-Body Controller that receives feedback from a state estimation module, based on either (i) external motion capture system or (ii) proprioceptive sensors.

First, we briefly describe the humanoid robot RH5 used in practical experiments. Second, we introduce the motion capture system and explain its application for tracking the position and orientation of the robot’s floating base. Next, we describe the state estimation approach based on proprioceptive sensors used in this work for comparison with our motion capture system. Finally, we present the Whole-Body Control framework used on the humanoid robot RH5. The interaction between these components is depicted in Figure 1.

### 2.1. Humanoid Robot RH5

The robot RH5 [11] is a 2 m tall, 62.5 kg humanoid driven by a hybrid combination of serial and parallel actuation modules. For example, the RH5 ankle joints are designed as parallel submechanisms with 2 degrees of freedom (DOF), which are arranged in series with the other leg actuation modules (see [14] for a comprehensive overview). The robot has 34 DOF and is equipped with various sensors, such as an inertial measurement unit, joint encoders, force-torque sensors, foot contact sensors and a stereo camera. For proprioceptive state estimation, we rely on the IMU sensor, joint encoders and foot contact sensors. The IMU model used here is part of the Xsens MTI-300 series of attitude and heading reference systems. The robot’s foot contact with the ground consists of 4 contact sensors located at the corners of each foot. Additionally, there is a 6 DOF force/torque sensor on each foot. In the parallel submechanism modules, an absolute encoder is installed in the independent joints and a relative encoder in the linear actuators, ensuring correct forward and inverse geometric mappings.

### 2.2. Motion Capture System

The motion capture system used for rigid body tracking consists of 3 Oqus 300+ Qualisys cameras connected to a Windows 10 computer. The Qualisys Track Manager software allows tracking and streaming of rigid body poses over an Ethernet connection. We stream rigid body data in real time without further pre-processing steps to the RoCK software framework [15], which runs on the robot’s main control PC (Ubuntu 18.04). The rigid body tracking has a frequency of 750 Hz and a variable latency of 2–4 ms.

System calibration is achieved by placing the calibration frame with the desired position and orientation in the cameras’ field of view. The calibration process is fast and accurate, with average residual values of less than 0.5 mm. A new system calibration is only required after repositioning the cameras in the workspace, which means that recalibration between successive experiments is not required. The cameras can be placed in any configuration in the room as long as the markers are not occluded.

**Figure 2:**
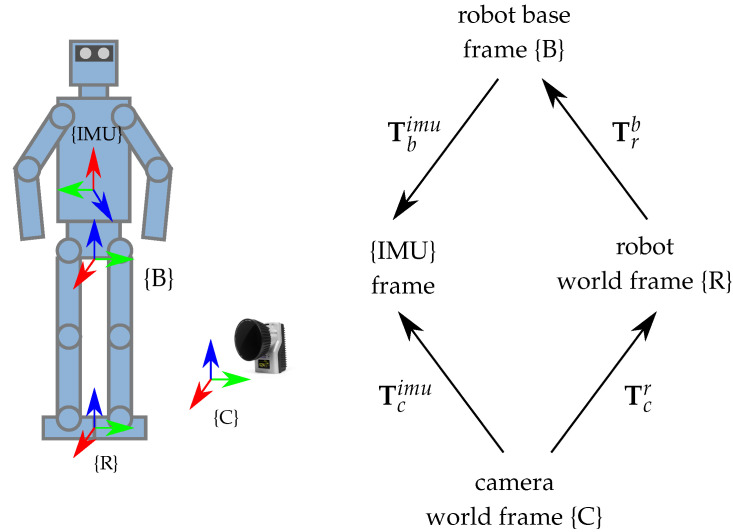
The coordinate frames used for robot floating base tracking are the camera world coordinate frame {C}, robot world coordinate frame {R}, robot base frame {B} and robot {IMU} frame. The corresponding transformation tree is depicted on the right hand side of the figure.

The motion capture system can be used to track any robotic platform and stream rigid body data in real time over the network. In our work, we use the motion capture system to track the robot {IMU} frame shown in Figure 2. In this way, we can retrieve the pose of the floating base of the humanoid and make a direct comparison with a state estimation approach based on IMU data.

**Figure 3:**
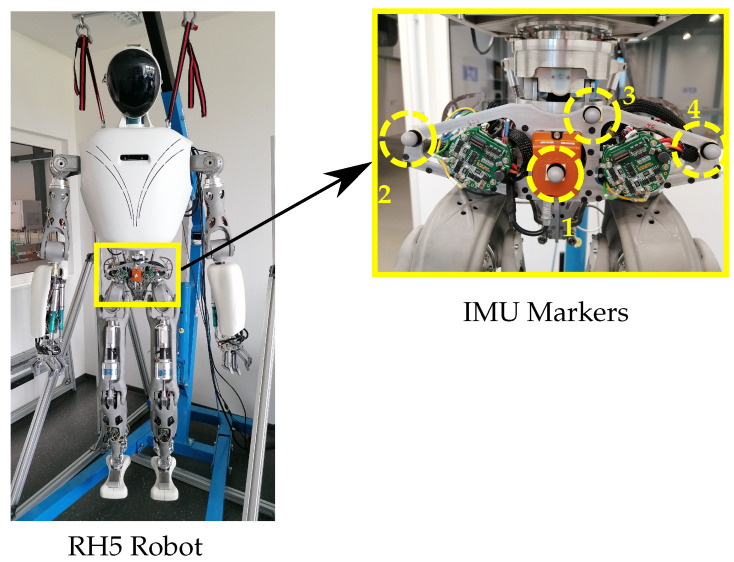
Four reflective markers are placed on the humanoid robot torso in order to track the robot IMU frame with a motion capture system.

Three markers are required to determine the position and orientation of a rigid body. For redundancy, we place 4 reflective markers on the robot torso as shown in Figure 3. This ensures better tracking performance in case of occlusions and robustness against outliers caused by reflective robot surfaces. We define the tracked IMU rigid body as follows: (i) the origin corresponds to *Marker 1* placed on the center of the IMU sensor, and (ii) the orientation is aligned with the right-handed robot {IMU} frame (*x*-up, *y*-right, *z*-forward).

Next, we apply a series of transformations to the IMU rigid body pose to (i) convert the tracked IMU pose to robot world coordinates using the camera-to-robot transformation $T_{c}^{r}$ and (ii) obtain the robot’s floating base pose $T_{r}^{b}$ in the robot world coordinate system. The camera world frame {C} and robot world frame {R} are shown along with all other relevant transformations in Figure 2.

The camera world frame {C} is defined during the calibration procedure of the motion capture system. The transformation of the robot world frame $T_{r}^{b,0}$ is defined as the projection of the base frame at the initial time t=0 onto the ground plane. The *z*-position component is set to zero and the orientation of the world frame is set to the initial orientation of the base frame. Then, the transformation between camera and robot world frame $T_{c}^{r}$ is computed using the transformation chain rule in Equation (1):

(1) $$T_{c}^{r}=T_{c}^{imu,0}\;{\left(T_{b}^{imu}\right)}^{-1}\;{\left(T_{r}^{b,0}\right)}^{-1},$$

where $T_{c}^{imu,0}$ is the tracked IMU rigid body pose in camera coordinates at time t=0 and $T_{b}^{imu}$ is the fixed IMU frame transformation with respect to the robot base frame.

Finally, we can recover the tracked floating base pose of the robot $T_{r}^{b,i}$ in robot world coordinates {R} at time t=i. For this purpose, we apply a series of transformations from (i) robot to camera frame ${\left(T_{c}^{r}\right)}^{-1}$, (ii) camera to IMU frame $T_{c}^{imu,i}$ at time t=i and (iii) IMU to floating base frame of the robot ${\left(T_{b}^{imu}\right)}^{-1}$ as shown in Equation (2):

(2) $$T_{r}^{b,i}={\left(T_{c}^{r}\right)}^{-1}\;T_{c}^{imu,i}\;{\left(T_{b}^{imu}\right)}^{-1}.$$

In Equation (2), the time index *i* denotes transformations of tracked rigid bodies, which are updated at every time step. The other transformations are fixed frames that are constant over time.

### 2.3. State Estimation

**Figure 4:**
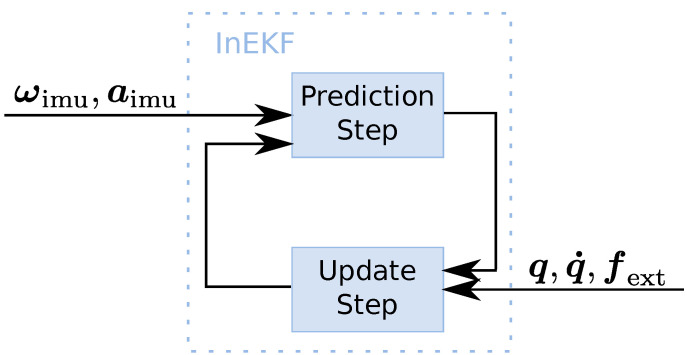
Prediction and update blocks of the InEKF proprioceptive state estimator.

The proprioceptive state estimator uses the invariant extended Kalman filter (InEKF) proposed by [13]. The filter fuses sensor information from IMU, leg odometry and foot contact sensors. The IMU linear acceleration $a_{\mathrm{imu}}$ and angular velocity $\omega_{\mathrm{imu}}$ data are used as input to the prediction step of the InEKF. The update step is performed based on leg kinematics $q,\dot{q}$ and foot contact information $f_{\mathrm{ext}}$ as shown in Figure 4.

The system state $X\in R^{\left(n+5)\times (n+5\right)}$ estimated by the InEKF is defined in Equation (3):

(3) $$X={\left[\begin{array}{cccccc} R & v & p & p_{C_{1}} & \ldots & p_{C_{n}}\\ 0_{1,3} & 1 & 0 & 0 & \ldots & 0\\ 0_{1,3} & 0 & 1 & 0 & \ldots & 0\\ \vdots & \vdots & \vdots & \vdots & \ddots & \vdots \\ 0_{1,3} & 0 & 0 & 0 & \ldots & 1 \end{array}\right]}_{\left(n+5),\;(n+5\right)},$$

where $R\in R^{3\times 3},v\in R^{3}$ and $p\in R^{3}$ represent the orientation, velocity and position of the robot’s floating base, and $p_{C_{i}}\in R^{3}$ represents the position of the *n* foot contact points.

In contrast to the standard EKF, the InEKF [16] takes advantage of Lie Group theory [17]. Lie Groups are collections of object symmetries, for instance, the collection of rotation matrices of a 3D object in space, known as the Special Orthogonal Group SO(3). Instead of using Jacobians to linearize the system, the InEKF operates on a linear vector space, namely the Lie algebra *g* of a given Lie Group G. The Lie algebra is defined as the tangent to the Lie Group manifold at the identity element. The mapping from the Lie algebra to the Lie Group is given by the exponential map exp in Equation (4), while the reverse mapping is provided by the logarithmic map log from Equation (5):

(4) $$\begin{array}{ccc} exp & :g\to G;\hat{\tau }\mapsto X=exp\left(\hat{\tau }\right) & \end{array}$$

(5) $$\begin{array}{cc} log & :G\to g;X\mapsto \hat{\tau }=log\left(X\right), \end{array}$$

where $\hat{\tau }$ is the estimated state on the Lie algebra and the X is the state represented on the matrix Lie Group manifold.

In the InEKF, the exponential map is used to update the state estimate and determine the exact error on the Lie Group manifold. The filter has strong convergence provided by the invariance properties of the Lie Group and allows linearization independent of the current system state. However, when the IMU accelerometer and gyroscope biases are added to the state matrix, it loses the group affine property required for a matrix Lie Group. This leads to an “Imperfect InEKF”, and the estimation error cannot be exactly retrieved anymore. Moreover, the filter still suffers from inertial drift, yaw unobservability and uncertainties in forwards kinematics due to leg flexibility.

These shortcomings of the proprioceptive state estimator may hinder the execution of complex and dynamic motions required for bipedal locomotion and affect the robot stability during single leg support phases and in challenging environments. Hence, state feedback through motion capture is proposed as an alternative for developing and testing new controllers for the humanoid robot RH5.

### 2.4. Whole-Body Control

To stabilize the robot during motions such as squatting and balancing, we use a velocity-based Whole-Body Control (WBC) framework (https://github.com/ARC-OPT/wbc, accessed on 11 December 2022) [12], which solves the following instantaneous quadratic program (QP):

(6) $$\begin{array}{cc} \min_{\dot{q}} & ∥\sum_{i}w_{i}\left(J^{i}\dot{q}-v_{d}^{i}\right){∥}_{2}\\ s.t. & J_{c}^{j}\dot{q}=0,\;j=1,\ldots ,K\\ \; & {\dot{q}}_{m}\leq \dot{q}\leq {\dot{q}}_{M}, \end{array}$$

where $\dot{q}\in R^{6+n}$ are the robot joint velocities, including 6-DOF virtual floating base, *n* number of robot joints, $v_{d}^{i}\in R^{6}$ is the desired spatial velocity for the *i*-th task, $J^{i}\in R^{6\times (6+n)}$ is the associated task Jacobian and $w_{i}\;\in \;R^{6}$ the task weights that control the priority of a task. The QP is subject to joint velocity limits ${\dot{q}}_{m},{\dot{q}}_{M}\in R^{6+n}$, as well as *K* rigid contact constraints, where $J_{c}^{j}\in R^{6\times (6+n)}$ is the contact Jacobian of the *j*-th contact point. The QP in Equation (6) can be solved using any standard QP solver, e.g., [18]. Robot tasks are specified by providing trajectories for $v_{d}^{i}$, for instance, by means of feedback controllers designed around the QP in Equation (6). For full pose control, this can be achieved as follows:

(7) $$v_{d}=v_{r}+K_{p}\left(\begin{array}{c} x_{r}-x\\ \theta {\hat{\omega }}_{r}^{a} \end{array}\right),$$

where $v_{r}\in R^{6}$ is the feed forward spatial velocity, $K_{p}\in R^{6\times 6}$ the feedback gain matrix, $x_{r},x\in R^{3}$ the reference and actual position of the robot and $\theta {\hat{\omega }}_{r}^{a}\in R^{3}$ the difference in orientation between actual and reference pose using a singularity-free representation [19].

**Figure 5:**
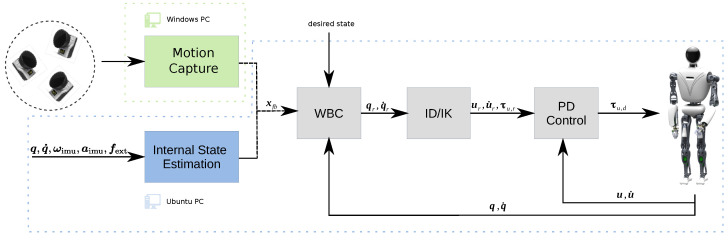
Control architecture to stabilize the desired robot motions.

The solution of Equation (6) is fed into an inverse dynamics solver [20] as shown in Figure 5, which not only computes the joint torques $\tau \in R^{n}$ for the entire robot, but also projects $q,\dot{q},\tau$ into the actuator space of the system, including all parallel kinematic mechanisms (PKM) of RH5. This avoids the usual mechanism-specific transformation of the solution to the actuators of each PKM, e.g., to the linear actuators in the RH5 ankle mechanism. As a result, we obtain the reference actuator positions, velocities and forces/torques $u,\dot{u},\tau_{u,r}\in R^{p}$, where *p* is the number of actuators in the robot. On actuator level, these are stabilized using a PD position controller with force/torque feed forward, which runs at 1 kHz, as shown in Equation (8):

(8) $$\tau_{u,d}=\tau_{u,r}+K_{d,u}\left({\dot{u}}_{r}-\dot{u}\right)+K_{p,u}\left(u_{r}-u\right),$$

where $K_{d,u},K_{p,u}\in R^{p\times p}$ are again diagonal feedback gain matrices.

The controller in Equation (7) is used to control the center of mass (CoM) and the orientation of the upper body of the humanoid robot, where the state feedback $x_{bf}$ of the robot’s floating base is provided by either (i) the external motion capture framework described in Section 2.2 or by using (ii) an internal state estimation approach as described in Section 2.3.

## 3. Results

In this section, we present experimental results obtained with the humanoid robot RH5. The robot is supposed to perform two different whole-body motions, namely squatting and balancing on one leg (please find the Supplementary Video S1 under https://www.youtube.com/watch?v=bqiBvVHf2i0, accessed on 11 December 2022). The two sets of experiments are performed using the whole-body controller in Section 2.4 with state feedback from motion capture system and proprioceptive state estimation, respectively. In the squatting experiments, tracking of the vertical motion of the robot is evaluated at two execution speeds of 10 and 16 s per squat. In the single leg balancing experiments, CoM and foot tracking are evaluated when the robot raises one leg at two different heights of 10 cm and 15 cm, respectively. At the beginning of each experiment, the robot is placed in its initial joint configuration and stands freely on the floor with both feet in contact with the ground. For safety reasons, a movable cord is attached to the robot’s torso, which is secured by a crane and neither restricts the robot’s movement nor affects its stability.

### 3.1. Squatting Experiment

**Figure 6:**
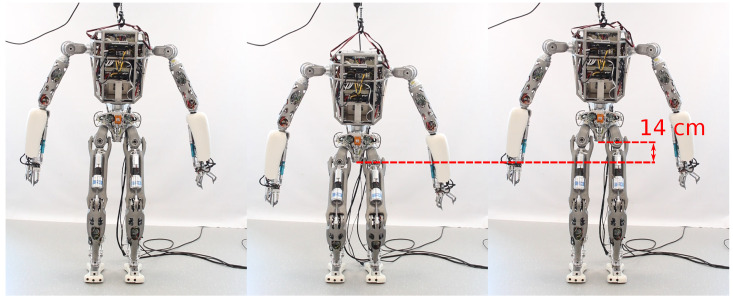
Time lapse of the humanoid robot RH5 performing squats with an amplitude of 14 cm.

In the squatting experiments, the floating base of the robot performs a translation along the vertical *z*-axis with a height difference of 14 cm, as shown in Figure 6. Using the whole-body controller described in Section 2.4, we constrain the feet to be in contact with the ground during motion execution. In addition, we split the squatting motion into two control tasks, namely the root task and the CoM task, as shown in Table 1. The root task is used to constrain the floating base of the robot to follow the desired vertical motion on the *z*-axis and minimize the lateral motion on the *y*-axis. The position of the floating base on the *x*-axis is not constrained to allow the root frame to move forward and backward if necessary, similar to the human squat. Furthermore, the CoM task is used to keep the ground projection of the robot CoM centered in the support polygon to enforce balance.

We performed two experiments with five squat repetitions each. The execution speed was constant in both scenarios, while the time interval for a squat varies, namely (i) experiment S1 with one squat per 16 s and (ii) experiment S2 with one squat per 10 s. In this way, we can evaluate the squat movement with and without a stabilization break between the movement direction changes. Both sets of experiments were successfully conducted using state feedback from (a) external motion tracking and (b) proprioceptive state estimation.

Due to the initial yaw angle unobservability of the proprioceptive state estimator, the reference frame of the estimator is arbitrarily rotated around the *z*-axis. We account for this rotation by adjusting the setpoints accordingly. To obtain a rotation-invariant error and compare the two state feedback approaches, i.e., MoCap and proprioceptive state estimation, we compute the 3D Euclidean space root-mean-square error (RMSE) between the desired and measured robot position, as shown in Equation (9):

(9) $$E_{c}\;=\;\sqrt{E_{c,x}^{2}+E_{c,y}^{2}+E_{c,z}^{2}}\;,$$

where $E_{c,x}$ and $E_{c,y}$ represent the CoM tracking error on the *x* and *y*-axis, $E_{c,z}$ represents the floating base tracking error on the *z*-axis, and $E_{c}$ represents the RMSE tracking error in Euclidean space. Since the calibration residuals of the motion capture system are less than or equal to ±0.5 mm, we evaluate the experimental data to an accuracy of 1 mm.

**Figure 7:**
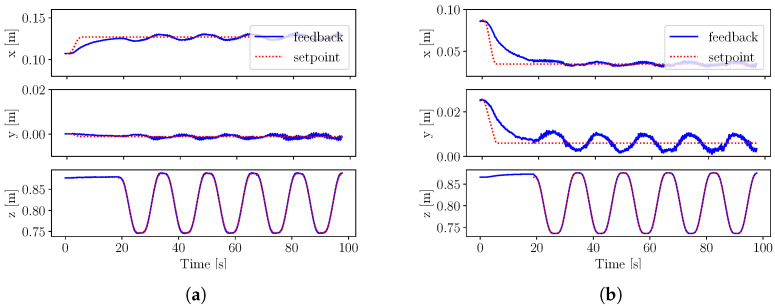
Squatting experiments S1, where the robot CoM position on *x* and *y*-axis and the floating base position on the *z*-axis are tracked by the whole-body controller using (**a**) motion capture state feedback and (**b**) proprioceptive state estimation. (**a**) Squatting motion using external motion capture state feedback with the respective RMSE as follows: $E_{c,x}=0.004$, $E_{c,y}=0.001$ and $E_{c,z}=0.001$. (**b**) Squatting motion using proprioceptive state estimation feedback with the respective RMSE as follows: $E_{c,x}=0.007$, $E_{c,y}=0.004$ and $E_{c,z}=0.001$.

The tracking results of experiment S1 are shown in Figure 7. We observe slightly better stability using external motion capture feedback compared to proprioceptive state estimation. As shown in Table 2, proprioceptive feedback is responsible for larger errors when the time interval between squats decreases in experiment S2. This shows that state estimation becomes less accurate during fast movements, whereas performance does not change significantly during squatting with external motion capture feedback.

### 3.2. One Leg Balancing Experiment

**Figure 8:**
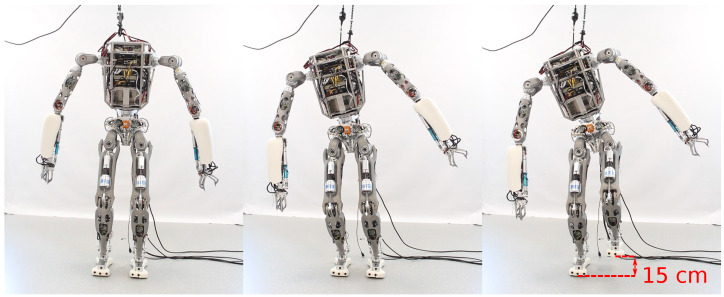
Time lapse of the humanoid robot RH5 balancing on the right leg, while raising the left leg at 15 cm above the ground.

During the balancing experiments, the robot starts with both feet in contact with the ground. From the double leg support phase, the robot switches to the single leg support phase by shifting the CoM to the right foot and raising the left foot to a 15 cm height, as shown in Figure 8.

We achieved this behavior using Whole-Body Control, with a Cartesian task for raising the left foot and a CoM task for constraining the position of the robot’s center of mass. To achieve human-like motion, both wrists are constrained by Cartesian tasks to keep them in front of the torso. Moreover, the contact constraint of the left foot is dynamically disabled during the lift-off phase and re-enabled during touchdown.

The setpoints for the tasks are generated using a trajectory interpolator and executed at joint level using the PD position controller in [20]. To enforce static balance of the robot, larger weights have been chosen for the *x* and *y*-axes of the CoM position with respect to the CoM vertical axis, as shown in Table 1.

We successfully performed experiments on balancing on one leg by tracking the robot’s floating base using (i) a motion capture system and (ii) proprioceptive pose estimation. We defined two scenarios, namely experiment B1 and B2, in which the vertical setpoint of the left foot reaches a height of 10 cm and 15 cm, respectively. In both experiments, the center of mass has been lowered by 4 cm on the *z*-axis to increase stability.

**Figure 9:**
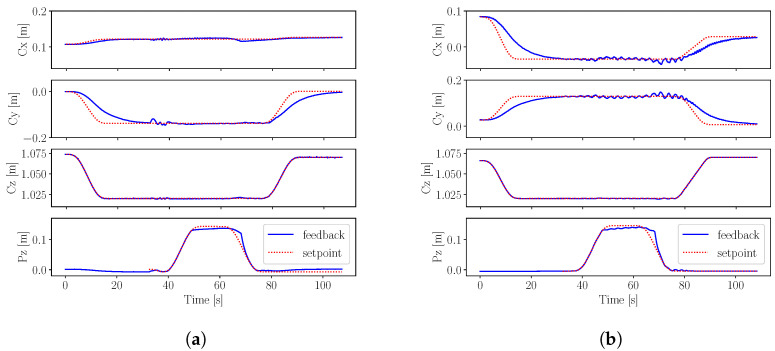
Single leg balancing experiments B2, where the robot CoM position $C_{x},C_{y},\;\mathrm{and}\;C_{z}$ on the *x*, *y* and *z*-axis, respectively, as well as the foot position $P_{z}$ on the *z*-axis are tracked by the whole-body controller using (**a**) motion capture state feedback and (**b**) proprioceptive state estimation. (**a**) One leg balancing using external motion capture state feedback with the respective RMSE as follows: $E_{c,x}=0.002$, $E_{c,y}=0.023$, $E_{c,z}=0.001$ and $E_{p}=0.008$. (**b**) One leg balancing using proprioceptive state estimation feedback with the respective RMSE as follows: $E_{c,x}=0.017$, $E_{c,y}=0.019$, $E_{c,z}=0.001$ and $E_{p}=0.008$.

The results of experiment B2 are shown in Figure 9. We notice larger oscillations on the *x* and *y*-axis when using proprioceptive state estimation feedback, as opposed to external motion capture feedback. In both balancing experiments with motion capture feedback, we observe stable single leg balancing, as summarized in Table 2.

## 4. Discussion

The experiments with the RH5 humanoid on squatting and single leg balancing compare two approaches for providing state feedback for Whole-Body Control, namely an external motion capture system and proprioceptive state estimation.

Proprioceptive state estimation provides fast state estimates relying only on proprioceptive sensors such as IMU, position readings from the joints and contact sensors. However, it suffers from yaw unobservability, and we apply an additional transformation to the desired COM trajectory to cope with the initial yaw estimation error. Moreover, the results show that both squatting and single leg balancing motions with proprioceptive state estimation feedback suffer from oscillations when the speed or complexity of the motion increases. This might be caused by the uncertainties in the “Imperfect InEFK” estimation, since the IMU biases from the state vector do not satisfy the matrix Lie group properties.

In contrast, the external motion capture system consists of cameras tracking reflective markers on the robot’s IMU frame. The employed motion capture system is able to provide accurate and fast state feedback to the whole-body controller with minimal setup and calibration efforts. The results show that external motion capture feedback contributes to more stable motions in the squatting and single leg balancing experiments. Due to its robustness and suitability for high-frequency closed-loop control, this method could enable the robot to execute more complex motions in the future, such as walking, climbing stairs and multi-contact tasks. Thus, external motion capture feedback can contribute to the development and testing of robot capabilities and Whole-Body Control algorithms.

## 5. Conclusions

Floating base state estimation plays a key role in bipedal locomotion of a humanoid robot since state estimation errors can affect the robot’s balance in double or single leg support phases. In this work, we show investigations on the use of external motion capture feedback for humanoid robot control and compare it with a state-of-the-art proprioceptive state estimation method. We perform two different whole-body motions with the humanoid robot RH5, namely squatting and single leg balancing and track the robot’s floating base using external cameras. We demonstrate that high-frequency external motion capture feedback can be reliably used for Whole-Body Control of humanoid robots and shows better stability than proprioceptive sensing, which is subject to noise and drift. As possible applications, external motion capture could be used both in industrial workspaces such as factories and in research laboratories in parallel with the development of better proprioceptive state estimation approaches to improve Whole-Body Control algorithms and explore the capabilities of humanoid robots.

In future work, we consider addressing possible MoCap errors such as outlier rejection and marker placement in order to increase performance. Moreover, fusion of proprioceptive state estimation and real-time motion capture data could reduce the state estimation drift and enable the robot to perform robust bipedal locomotion or other multi-contact tasks.

## Figures and Tables

**Table 1:** Task weights used in the whole-body controller for squatting and single leg balancing.

Experiment	Task	Weights					
		x	y	z	$\theta_{x}$	$\theta_{y}$	$\theta_{z}$
Squatting	CoM	6	6	0	-	-	-
	Root	0	1	1	1	1	1
Balancing	CoM	6	6	1	-	-	-
	Feet	1	1	1	1	1	1
	Wrists	1	1	0	0	0	0

**Table 2:** Tracking error of the robot CoM position $E_{c}$ along the three axes and foot position on the *z*-axis ($E_{p}$) during the squatting and single leg balancing experiments. The highlighted values represent the smallest CoM and foot position tracking errors for every set of experiments.

Experiment	State Feedback	RMSE [m]				
		$E_{c}$	$E_{c,x}$	$E_{c,y}$	$E_{c,z}$	$E_{p}$
S1 (16 s)	MoCap Tracking	**0.004**	0.004	0.001	0.001	-
	State Estimation	0.008	0.007	0.004	0.001	-
S2 (10 s)	MoCap Tracking	**0.004**	0.004	0.001	0.002	-
	State Estimation	0.027	0.010	0.025	0.001	-
B1 (10 cm)	MoCap Tracking	**0.025**	0.002	0.025	0.001	0.006
	State Estimation	0.026	0.018	0.018	0.001	**0.002**
B2 (15 cm)	MoCap Tracking	**0.023**	0.002	0.023	0.001	0.008
	State Estimation	0.026	0.017	0.019	0.001	0.008

## Data Availability

Not applicable.

## Supplementary Materials

The following supporting information can be accessed at: https://www.youtube.com/watch?v=bqiBvVHf2i0, Video S1: Experimental Investigations into Using Motion Capture State Feedback for Real-Time Control of a Humanoid Robot.
